# Health intelligence: how artificial intelligence transforms population and personalized health

**DOI:** 10.1038/s41746-018-0058-9

**Published:** 2018-10-02

**Authors:** Arash Shaban-Nejad, Martin Michalowski, David L. Buckeridge

**Affiliations:** 1 Department of Pediatrics, University of Tennessee Health Science Center – Oak Ridge National Laboratory (UTHSC-ORNL) Center for Biomedical Informatics, Memphis, TN USA; 2 School of Nursing, University of Minnesota, Minneapolis, MN USA; 3 Department of Epidemiology, Biostatistics and Occupational Health, McGill University, Montreal, QC USA

**Keywords:** Population screening, Research management

## Abstract

Advances in computational and data sciences for data management, integration, mining, classification, filtering, visualization along with engineering innovations in medical devices have prompted demands for more comprehensive and coherent strategies to address the most fundamental questions in health care and medicine. Theory, methods, and models from artificial intelligence (AI) are changing the health care landscape in clinical and community settings and have already shown promising results in multiple applications in healthcare including, integrated health information systems, patient education, geocoding health data, social media analytics, epidemic and syndromic surveillance, predictive modeling and decision support, mobile health, and medical imaging (e.g. radiology and retinal image analyses). Health intelligence uses tools and methods from artificial intelligence and data science to provide better insights, reduce waste and wait time, and increase speed, service efficiencies, level of accuracy, and productivity in health care and medicine.

## Population health intelligence

Public health authorities and researchers collect data from many sources and analyze these data together to estimate the incidence and prevalence of different health conditions, as well as related risk factors. To present a coherent portrait^1^ of population health, AI methods extract health and non-health data at different levels of granularity, harmonize and integrate information about populations and communities with evidence about the epidemiology and control of chronic diseases.

### Sociomarkers

It is widely recognized that many of the elements that shape the health and well-being of individuals and communities have their roots outside the conventional healthcare system. Recent methodological developments in AI enable multi-level modeling to combine individual-level data with sociomarkers, the measurable indicators of social conditions, at the group-level to improve disease surveillance, disease prediction, and implementation and evaluation of population health interventions. Shin et al.^2^ shows how the social determinants of health can be used to predict and identify pediatric asthma patients at risk of hospital revisits.

### Mobile health

Increasing mobile connectivity and the popularity of wearable devices as well as advances in technologies such as health IoT (the use of Internet of Things to facilitate medical device integration) have provided opportunities for clinicians and public health researchers to better understand the physiological variability in individuals and populations to diagnose and deliver care, and to better plan to implement preventive and therapeutic measures. Chunara et al.^3^ discusses how to track health seeking behaviors and measure public and private sector facility utilization during an Ebola outbreak via mobile phones and short messaging service (SMS).

### The internet and health

People, specifically teens and youth, spend a significant amount of their time interacting with or consuming many types of media, with the Internet as their primary source of health information.^4^ The internet has expanded public health research beyond its traditional realm.^5^ Tracking of Internet-based health indicators complements other surveillance methods collecting data from clinical systems and registries. Advances in intelligent web-based applications and online smart devices and social media platforms assist public health practitioners and researchers in disease surveillance and epidemic detection, behavior monitoring, and public health communication and education.

## Personalized health intelligence

Health intelligence is a basic block and major driver for initiatives such as precision medicine.^6^ Personalized healthcare is an approach for disease management that considers individual variability in environment, lifestyle and genes for each person. Initiatives such as the National Institute of Health (NIH) All of Us Research Program^7^ aim to pioneer a new model of patient-powered research to develop health care solutions that make the best decisions to prevent or treat a disease, predict epidemics, and improve the quality of life. To tackle and overcome several issues in personalized healthcare, information technology must evolve to improve communication, collaboration, and teamwork between patients, their families, healthcare communities, and care teams involving practitioners from different fields and specialties. For example, shared decision making is advocated for its increase of patient engagement and therapy compliance, improvement of health outcomes, and alignment of care with patient values.^8^

Artificial intelligence is currently used to process personalized data, to elicit patient preferences, to help patients (and families) to participate in the care process, to help physicians use this participation to provide high quality and efficient personalized care by personalizing “generic” therapy plans and connecting patients with information beyond those available within their care setting.^9^ Clinicians and public health practitioners can use the technology to deliver tailored therapy or interventions based on the best evidence to maintain high-quality patient care and create healthier communities. For example, Arandjelovic et al.^10^ demonstrate the use of machine learning to improve the accuracy of Stage II colorectal cancer prognosis.

## Challenges and opportunities

Despite the wide-spread use of intelligent applications in healthcare there remain challenges to their adoption. The acceptance of technology—especially for diagnostics in clinical setting, concerns related to scalability, data integration and interoperability, security, privacy and ethics of aggregated digital data are just some of the examples of the challenges ahead. For example, the early adaption of AI methods in online social media analytics revealed some ethical challenges that can potentially undermine the privacy and autonomy of individuals and cause stigmatization.^11^ Additionally, patient complexity is increasing with the average life expectancy in the US on the decline.^12^ Baby boomers are aging (20% of the 65+ population by 2029) and multi-morbidity affects 60% of this population and is associated with over twice as many patient-physician encounters. Social and behavioral contexts play critical roles in the management of these increasingly complex patients and as such need to be key components of technology-based solutions. Despite their limitations, AI tools and techniques that are still in their infancy already provide substantial benefits in providing in-depth knowledge on individuals’ health and predicting population health risks, and their use for medicine and public health is likely to increase substantially in the near future.
